# Discomfort and agitation in older adults with dementia

**DOI:** 10.1186/1471-2318-7-27

**Published:** 2007-11-22

**Authors:** Isabelle Chantale Pelletier, Philippe Landreville

**Affiliations:** 1 School of Psychology, Université Laval, Québec, Canada

## Abstract

**Background:**

A majority of patients with dementia present behavioral and psychological symptoms, such as agitation, which may increase their suffering, be difficult to manage by caregivers, and precipitate institutionalization. Although internal factors, such as discomfort, may be associated with agitation in patients with dementia, little research has examined this question. The goal of this study is to document the relationship between discomfort and agitation (including agitation subtypes) in older adults suffering from dementia.

**Methods:**

This correlational study used a cross-sectional design. Registered nurses (RNs) provided data on forty-nine residents from three long-term facilities. Discomfort, agitation, level of disability in performing activities of daily living (ADL), and severity of dementia were measured by RNs who were well acquainted with the residents, using the Discomfort Scale for patients with Dementia of the Alzheimer Type, the Cohen-Mansfield Agitation Inventory, the ADL subscale of the Functional Autonomy Measurement System, and the Functional Assessment Staging, respectively. RNs were given two weeks to complete and return all scales (i.e., the Cohen-Mansfield Agitation Inventory was completed at the end of the two weeks and all other scales were answered during this period). Other descriptive variables were obtained from the residents' medical file or care plan.

**Results:**

Hierarchical multiple regression analyses controlling for residents' characteristics (sex, severity of dementia, and disability) show that discomfort explains a significant share of the variance in overall agitation (28%, *p *< 0.001), non aggressive physical behavior (18%, *p *< 0.01) and verbally agitated behavior (30%, *p *< 0.001). No significant relationship is observed between discomfort and aggressive behavior but the power to detect this specific relationship was low.

**Conclusion:**

Our findings provide further evidence of the association between discomfort and agitation in persons with dementia and reveal that this association is particularly strong for verbally agitated behavior and non aggressive physical behavior.

## Background

Dementia is not an inevitable consequence of ageing but the risk of dementia increases sharply with advancing age and prevalence is expected to increase dramatically over the coming decades [1]. Dementia features an alteration of memory and at least one other cognitive disorder such as aphasia, agnosia, apraxia or a disturbance in executive functioning [2]. Various etiologies are related to dementia, such as strokes, head trauma, Parkinson's disease, and substance abuse. Yet Alzheimer's disease is considered the most widespread form of all senile dementias, representing more than half of all cases [3]. The onset of dementia of the Alzheimer's type (DAT) is gradual and involves continuing cognitive decline [2].

In addition to cognitive symptoms, persons with dementia often present behavioral and psychological symptoms which may increase their suffering, be difficult to manage by caregivers, and precipitate institutionalization [4]. Behavioral symptoms of dementia include wandering, screaming, and hitting, while psychological symptoms include hallucinations, delusions, and depression. Between 50 and 90% of dementia patients present with behavioral or psychological symptoms [5]. The term "agitation" is often used in reference to behavioral symptoms associated with dementia [6]. Agitation was originally defined as any inappropriate verbal, vocal or motor activity which, according to an outside observer, does not result directly from the needs or the confusion of the agitated person [7]. Behavior which constitutes agitation can be broadly classified as aggressive vs non-aggressive and physical vs verbal [8]. A factor analysis of a measure of agitation used with nursing home residents produced three factors which make it possible to distinguish various forms of agitation [9]: aggressive behavior (AB, e.g., hitting), non-aggressive physical behavior (NAPB, e.g., pacing), and verbally agitated behavior (VAB, e.g., complaining).

The specific determinants of agitation remain unclear [6]. Predisposing factors may include gender, personality, poor health, functional impairment of activities of daily living, as well as cognitive and neurological deterioration [10,11]. Other factors may precipitate the occurrence of agitation and include the characteristics of the physical and social environment (e.g., too much noise, not enough social interaction) as well as physical needs such as hunger, thirst, and discomfort. Some of these variables, such as sex, the severity of cognitive impairment and the level of dependence in performing activities of daily living, are well documented in the literature in terms of their relationship with different types of agitation. For example, males are more likely to be aggressive than females, NAPB is more likely to be manifested by persons who are more cognitively impaired, and VAB tends to be exhibited by persons who are more functionally impaired [8]. Other variables, such as hunger and light intensity, may be associated with agitation but this association remains hypothetical [12-14].

Several studies have examined environmental or contextual determinants of agitation [15,16]. Considerably less attention has been devoted to internal states which may also trigger difficult behaviors. Discomfort, which is defined as a negative emotional or physical state subject to variation in response to internal or environmental conditions [17], may act as an internal factor which precipitates the occurrence of agitation [10,12]. Persons suffering from dementia may behave in ways that are disruptive to those around them but which, for these patients, serve to communicate the discomfort they feel. For example, a patient suffering from moderate dementia and who gradually becomes aphasic may revert to shouting, emit odd noises, become unruly or hit those around him to let them know he feels pain during dressing and bathing. Various studies have shown the importance of discomfort in persons suffering from dementia [18-20] and a majority of patients must deal with painful acute or chronic ailments such as cancer, depression, cardiovascular disease and musculo-skeletal disorders [21].

We searched the literature (PsycINFO, CINAHL, Ageline, and Medline databases) and the reference list of selected papers for empirical studies of the relationship between discomfort and agitation in dementia. Two such studies were found. The first study, by Buffum et al. [20], has shown that more discomfort is associated with more agitation. However, the authors considered agitation as a single construct without considering its subtypes (i.e., AB, NAPB and VAB). Given that the importance of the relationship between different factors varies according to these subtypes, [8,11,12], discomfort may be more strongly related to certain subtypes of agitation than others. In their review of the literature, Cohen-Mansfield and Deutsch [8] indicated that all subtypes of agitation may be associated with discomfort. However, the available evidence strongly suggests this association in the case of VAB whereas the reasons for both AB (e.g., cognitive impairment, personality style, unsuccessful communication with caregivers) and NPAB (e.g., need for exercise or stimulation, performing previous roles) appear more diversified. In the second study, Young [22] used the Cohen-Mansfield Agitation Inventory (CMAI) [9] and reported low but significant positive correlations between discomfort and both overall agitation and AB. Unfortunately, these results are difficult to interpret because it is unclear whether the original 29-items version or a shorter 14-items version of the CMAI was used and what scale was used for rating each item. Moreover, the exact items used in calculating scores for each agitation subtype are not reported. Two additional scores of overall agitation, obtained from the Functional Abilities Checklist [23] and the Minimum Data Set, were also correlated with discomfort.

The goal of this study is to document the relationship between discomfort and agitation (including agitation subtypes) in older adults suffering from dementia. The following hypotheses were tested: (a) the frequency of overall agitation is related positively to the degree of discomfort, and (b) the degree of discomfort is related positively to frequency of VAB. To ensure more accurate results, sex, the severity of dementia and disability in performing activities of daily living, three variables whose relationship with agitation is well documented in the literature, were measured for control purposes.

## Methods

### Participants

This correlational study used a cross-sectional design. Thirteen registered nurses (RNs) from three long-term care facilities provided data on forty-nine residents. The RNs were all women, had a mean of 15 years of education and 22 years of experience working with older adults.

Residents were selected by the RNs according to the following criteria: (a) being at least 65 years old, (b) having a diagnosis of dementia documented by the medical file (all had a diagnosis of DAT), and (c) having been living in the same facility for at least three months. Residents suffering from delirium or any form of psychosis were excluded. Fifty-five residents were initially selected but data could not be collected for six of them because a respondent was not available during the data collection period. The final sample therefore included 49 residents, which is near the sample size estimate of 50 indicated by a power analysis for Pearson correlation coefficient (*r*) conducted prior to the study. This sample size estimate was obtained for a power of 0.80, an alpha level of 0.05, and an effect size of 0.40 determined from the correlation obtained between discomfort and overall agitation by Buffum et al. [20]. An effect size of 0.40 is halfway between medium (0.30) and large (0.50) effect size conventions for *r *proposed by Cohen [24]

### Measures

#### Descriptive variables

The following information was drawn from the resident's medical file and care plan: date of birth, sex, date of admittance to the facility, medical conditions, as well as diagnosis and type of dementia. Analgesics taken on a daily basis were also recorded for control reasons because these can influence the experience of discomfort. RNs, who filled out questionnaires for the residents, were also asked to provide descriptive information (gender, years of education, and years of experience working with older adults).

#### Severity of dementia

The level of cognitive impairment was measured using the Functional Assessment Staging (FAST) developed by Reisberg and colleagues [25,26]. The scale comprises seven stages measuring function loss associated with cognitive deterioration. Each resident was therefore given a score between 1 and 7 where 1 refers to a normal cognitive function and 7 to severe dementia with very severe cognitive impairment. Intraclass correlation coefficients indicate good test-retest reliability (0.86) and interrater agreement (0.87) [27]. Previous studies have indicated strong concurrent validity between FAST and various psychometric and mental status assessments [26,27]. For example, Sclan and Reisberg [27], found that increasing functional disability assessed by FAST is significantly related to decreasing cognitive functioning (*r *= -0.79).

#### Disability

The level of disability in performing activities of daily living (ADL) was measured using a subscale of the Functional Autonomy Measurement System (SMAF) [28,29]. This ADL subscale helps measure the level of impairment for five activities (feeding, washing, dressing, personal hygiene and using the bathroom), as well as urinary and fecal incontinence. Each item is evaluated using a four-point Likert-type scale where 0 indicates that the person is autonomous while -3 denotes dependency. Hébert and colleagues have found that the SMAF has acceptable interrater reliability (mean Cohen's weighted kappa of 0.75) and that the scale can be reliably used by both nurses and social workers in either community or institutional settings [28,29]. Results obtained by Desrosiers et al. show an intraclass correlation coefficient of 0.95 for test-retest and 0.96 for interrater reliability [30]. Concurrent validity is supported by a strong correlation between SMAF scores and the amount of nursing time required for care (*r *= 0.88) [28].

#### Agitation

Agitation was evaluated using the French version of the Cohen-Mansfield Agitation Inventory (CMAI) [31,32]. The frequency of all 29 items of the CMAI is evaluated for the previous two weeks using a Likert-type scale and scores given for each item varies from 1 ("never") to 7 ("several times per hour"). For each resident, an overall agitation (OA) score as well as a score for each one of the three factors of the CMAI (AB, NAPB, and VAB) were calculated by summing across items. These factors have been identified for the French version of the CMAI [31] and the corresponding items are very similar to those of the factors of the original version [9]: AB (6 items: hitting, grabbing, kicking, scratching, pushing, and spitting), NAPB (6 items: pacing, trying to get to a different place, handling things inappropriately, hoarding, hiding, general restlessness), and VAB (6 items: complaining, repetitious sentences or questions, negativism, constant requests for attention, cursing or verbal aggression, screaming). Deslauriers et al. [30] have demonstrated the interrater reliability (*r *= 0.72), test-retest reliability (*r *= 0.72), internal consistency (Cronbach's alpha from 0.75 to 0.77), concurrent validity (*r *= 0.74) and construct validity of the French version of the CMAI completed by nurses.

#### Discomfort

The Discomfort Scale for patients with Dementia of the Alzheimer Type (DS-DAT) [17] is composed of nine behavioral indicators of discomfort determined following interviews with caregivers working with persons suffering from dementia. Thus, the authors of the scale gave particular attention to content validity by identifying the kinds of behavior most frequently associated with a sign of discomfort in this population. The nine indicators are (a) noisy breathing, (b) negative vocalization, (c) content facial expression, (d) sad facial expression, (e) frightened facial expression, (f) frown, (g) relaxed body language, (h), tense body language, and (i) fidgeting. Each item comes with a list of observable forms of behavior which helps evaluators observe and record the signs of discomfort as objectively as possible. This tool makes it possible to evaluate the frequency (from 0 to ≥3), intensity (high or low) and duration (long or short) of the nine indicators associated with discomfort, as perceived by the observer, in the course of an observation period usually lasting five minutes. The level of discomfort is then derived from the value attributed to these three components. Each of the nine items is evaluated independently on a scale from 0 ("no observed discomfort") to 3 ("high level of observed discomfort"). Content validity was insured by initially generating twenty-six items from interviews with nursing staff from inpatient Alzheimer units and then retaining eighteen items rated by independent nurses holding advanced nursing degrees as relevant with an operational definition of discomfort (the number of items was later reduced to nine). The internal consistency of the DS-DAT has been evaluated in two studies [17,22] with results showing Cronbach's alpha varying from 0.74 to 0.89. The interrater reliability (*r *varying between 0.61 and 0.98) and test-retest reliability (*r *= 0.6) have also been verified [17,22,33]. Nurses acted as raters in studies reporting the psychometric properties of the DS-DAT [22,33].

### Procedure

RNs working on the residents' units were asked to collaborate in this study. A group meeting was called with the principal investigator (ICP) to explain the study to the RNs, obtain their consent, and provide them with information so that they would be able to identify residents matching the study criteria based on each resident's file. The RNs were also given a presentation to demonstrate how to complete each of the scales used in the study. Because the administration and scoring of the DS-DAT is complex, a large portion of the meeting was devoted to demonstrating how to use this tool. Each item of the scale was explained in detail, case examples were presented, and staff members had the opportunity to practice scoring. These instructions included Hurley et al.'s [17] recommendations regarding the timing of the assessment.

The DS-DAT, the CMAI, the ADL subscale of the SMAF and the FAST were completed by the RN most familiar with each resident. They were given two weeks to complete and return all scales (i.e., the CMAI was completed at the end of the two weeks and all other scales were answered during this period). The principal investigator could be contacted during this period to answer questions or provide additional information regarding the procedure.

Staff members were the participants in this study. Indeed, the main variables reflect their perception of selected residents. The residents themselves did not participate directly in the data collection. Staff members did provide the researchers with factual information obtained from the residents' file to which they had authorized access as part of their work. Prior to the study, the researchers were granted permission to obtain this information by the Professional Services Director of each participating facility. Residents' rights were protected by concealing their identity (a code was used instead of their name on each document related to the study). Université Laval's Research Ethics Committee approved this project.

## Results

Data analysis was performed using SPSS software, version 11.0 for Windows, on an Intel Pentium 4 computer with Microsoft Windows XP, Home Edition, Version 2002, as the operating system.

Residents characteristics are shown in Table 1. It should be noted that the sample was mostly comprised of women and that the majority of residents had a medical condition. In addition, nearly 60% of residents took an analgesic on a daily basis. For two of the 28 residents taking analgesics, the prescription was to take this medication if needed (prn) while the remaining residents took regularly scheduled analgesics at least once a day. This information suggests that an important proportion of the residents experienced chronic pain. Most residents were rated in the three last stages of the FAST which indicates that the cognitive functioning of most residents was highly impaired. The mean score on the SMAF (-15.5) was near the lowest score possible (-21), indicating that these residents were very dependent on the nursing staff.

**Table 1:** Residents characteristics

Characteristics	Percentage	*m*	Min, Max	*SD*
Sex				
Male	10.2			
Female	89.8			
Age (years)		82.7	67, 98	7.8
Duration of institutionalization (months)		38.7	3, 198	37.9
Number of medical conditions		5.2	1, 13	2.6
Daily administration of an analgesic	57.2			
Stage of dementia				
1	0.0			
2	0.0			
3	4.1			
4	2.0			
5	16.3			
6	53.1			
7	24.5			
Disability in performing ADL		-15.5	-1, -21	5.8
Agitation				
OA		41.5	29, 90	14.2
AB		7.2	6, 18	2.5
NAPB		10.0	6, 29	5.5
VAB		10.7	6, 32	6.1
Discomfort		4.2	0, 14	3.5

*Note*. OA = Overall agitation; AB = Aggressive behavior; NAPB = Non aggressive physical behavior; VAB = Verbally agitated behavior.

Table 2 shows the Pearson correlation coefficients (*r*) between each score of agitation, discomfort, and the remaining descriptive variables. Sex was coded as follows: men = 1 and women = 2. Positive and significant correlation coefficients are observed between discomfort and each of the following agitation scores: OA, NAPB and VAB. No other correlation coefficient was significant. The power to detect significant correlation coefficients with our data (*n *= 49) was examined using GPower software, version 3.0. for Windows [34]. Post hoc power analyses were conducted for t tests with alpha level fixed at 0.05. Power analyses for OA and VAB were for one-tailed tests because of our hypotheses regarding these variables, while analyses for two-tailed tests were done for NAPB and AB. Power was high for tests using OA (0.99; effect size = 0.55), VAB (0.99; effect size = 0.53), and PNAB (0.95; effect size = 0.47) but the test using AB had low power (0.37; effect size = 0.23).

**Table 2:** Correlation coefficients between agitation scores, discomfort and the other descriptive variables

	Sex	Severity of dementia	Disability in performing ADL	Level of discomfort
OA	-0.25	-0.00	0.04	0.56***
AB	-0.08	.014	-0.16	0.23
NAPB	-0.28	-0.10	0.06	0.47**
VAB	-0.24	-0.13	0.21	0.54***

*Note*. OA = Overall agitation; AB = Aggressive behavior; NAPB = Non aggressive physical behavior; VAB = Verbally agitated behavior.

**p < 0.01

***p < 0.001

Hierarchical multiple regression analyses were next performed to specify the relationship between discomfort and each agitation score (except AB) while statistically controlling for sex, severity of dementia and disability in performing ADL. For each analysis, these three last variables were introduced in a first block of the regression (Step 1), followed by the level of discomfort at the next step (Step 2). Results are shown in Table 3 which presents variance explained (R^2^) at Step 1, incremental variance (Δ*R*^2^) as a result of entering discomfort at Step 2, and total variance. The other statistics presented are the unstandardized (*B*) regression coefficients, the standard error of regression coefficients (*SE B*), and the standardized beta (*β*) regression coefficients at Step 2. The estimated *β *coefficients indicate the relative contribution of each independent variable in the prediction of the dependent variable (agitation). As shown, discomfort contributed significantly to the prediction of the variance of OA, NAPB and VAB, beyond the residents' other characteristics that were statistically controlled in each regression equation. The variance of the different agitation scores specifically attribuable to discomfort (Δ*R*^2^) varied between 18% and 30%. Further, estimated standardized beta coefficients show that only discomfort carries significant weight in the regression equation for OA, NAPB and VAB.

**Table 3:** Summary of hierarchical multiple regression analysis for variables predicting agitation, non aggressive physical behavior, and verbally agitated behavior

Variable	*B*	*SE B*	β
Overall agitation			
Sex	-4.17	6.01	-0.09
Severity of dementia	.94	3.09	0.06
Disability (ADL)	.40	0.50	0.17
Level of discomfort	2.20	0.52	0.55***
*R*^2 ^for Step 1 = 0.06, *F*(3,45) = 0.98 (p = 0.648) Δ*R*^2 ^for Step 2 = 0.28, *F*(1,44) = 18.21*** R^2 ^Total = 0.34, *F*(4,44) = 5.57***			
Non aggressive physical behavior			
Sex	-3.00	2.46	-0.17
Severity of dementia	-1.14	1.27	-0.19
Disability (ADL)	-0.04	0.21	-0.05
Level of discomfort	0.70	0.21	0.45**
*R*^2 ^for Step 1 = 0.09, *F*(3,45) = 1.55 (p = 0.214) Δ*R*^2 ^for Step 2 = 0.18, *F*(1,44) = 10.89** R^2 ^Total = 0.28, *F*(4,44) = 4.14**			
Verbally agitated behavior			
Sex	-0.98	2.51	-0.05
Severity of dementia	0.57	1.29	0.09
Disability (ADL)	0.38	0.21	0.36
Level of discomfort	0.99	0.22	0.57**
*R*^2 ^for Step 1 = 0.09, *F*(3,45) = 1.45 (p = 0.241) Δ*R*^2 ^for Step 2 = 0.30, *F*(1,44) = 21.04*** R^2 ^Total = 0.38, *F*(4,44) = 6.83***			

*Note*. Sex, severity of dementia and disability were introduced in a first block of the regression (Step 1), followed by the level of discomfort at the next step (Step 2). Statistics shown for each variable are those at Step 2.

**p < 0.01

***p < 0.001

## Discussion

The purpose of this study was to document the relationship between discomfort and the various types of agitation in older adults suffering from dementia. As hypothesized, our results show a positive and significant relationship between the degree of discomfort and the frequency of overall agitation. This confirms results obtained previously by Buffum and colleagues [20] and Young [22]. Our results further demonstrate that the relationship with discomfort varies according to the type of agitation. Also as hypothesized, we found that the degree of discomfort is associated both positively and significantly with VAB. Furthermore, our results show a positive and significant relationship with NAPB. No significant relationship was observed between discomfort and aggressive behavior but it should be noted that the power to detect this specific relationship was low and that the correlation coefficient obtained from our data (0.23) is similar to the one reported by Young (0.25).

Various authors contend that discomfort acts as an internal factor which precipitates the occurrence of agitation [10,12]. VAB possibly acts as a means of communicating the patient's discomfort [8,35]. Matteau and colleagues [35] have found that patients that display VAB also present more language difficulties. Through behaviors such as complaining and screaming, patients may attempt to attract their caregivers' attention in the hope that they will provide them some relief. The relationship between discomfort and NAPB was unexpected and is less clear. It is possible that several types of disruptive behavior related to this type of agitation, such as wandering and pacing, result from the discomfort experienced by the patient. For example, a patient who feels sad and depressed because he or she is homesick may attempt to leave his residential facility to reduce this discomfort [8].

Algase and colleagues [12] have suggested that behavioral problems associated with dementia result from unmet needs which the patient expresses using his remaining abilities. Given our results, the need for comfort appears as one such need. From a practical point of view, the occurrence of agitation deserves particular attention on the part of caregivers since it may communicate discomfort. Identifying and treating the cause of this discomfort may help reduce agitation. Kovach and colleagues [36] have recently reported findings which support this assertion. In their study, nursing home residents with dementia were treated using the Serial Trial Intervention which selects an appropriate treatment based on physical and affective needs assessments. Compared to a control group, the treated group had less discomfort and more frequently had behavioral symptoms return to baseline. Barton and colleagues [37] have suggested a similar hierarchical approach to the management of inappropriate vocalization.

The mean score on the DS-DAT in our sample (4.2) is lower than mean scores found in some studies [17,20] but similar to that reported by other researchers [22,33]. For example, a mean score of 4.64 was reported by Miller et al. [33] when their participants were assessed in situations of unlikely discomfort (the equivalent of what Hurley et al. [17] refer to as *baseline discomfort*). Also, the range of scores on the DS-DAT reported in Table 1 is broad (min to max: 0 to 14), suggesting that the DS-DAT was sensitive to various levels of discomfort in our sample.

The scores on the CMAI in our sample also deserve some comments. Looking at other's work, we found that CMAI scores are reported for clinical samples in which participants are selected because they present agitation [38,39]. These scores are higher than for the participants in our study who were not selected on the basis of agitation. For example, mean CMAI total scores vary between 65 and 78 across different samples in which participants present at least mild behavioral symptoms [39] compared to 41 in our sample. However, this mean score is similar to what we found previously in a separate but similar sample (mean scores of 42.6 and 40.7 at two separate times) [32]. Although there is no official cutoff for agitation on the CMAI, the range of scores (min to max: 29 to 90) is quite broad and suggests that some participants presented some agitation while others did not.

Limitations to the generalization of our findings are the relatively small sample size and the fact that the data were compiled only during the day shift. Some authors have indicated that the different types of agitation occur at different periods of the day [40,41]. It is unclear whether the same factors are equally important contributors to agitation at different periods of the day and this should be investigated. Another limitation is that it is uncertain whether the FAST is valid and reliable when used by nurses. However, it should be noted that the FAST is widely utilized by healthcare professionals of various backgrounds and requires minimal training because of its' face validity [42]. Other limitations have to do with the DS-DAT and have been pointed out in detail elsewhere [43]. This measure of discomfort provides no specific information as to the nature and origin of this experience. Discomfort is a broad concept referring to a negative emotional or physical state. Various conditions, including pain, distress, depression, loneliness, lack of stimulation, and lack of sleep can contribute to discomfort. Identifying which of these sources of discomfort play a role in agitation is essential for selecting appropriate treatment. Future studies, therefore, should identify the determinants of discomfort that are related to agitation in dementia patients and compare findings across different periods of the day. Finally, we did not check reliability because of restrictions in the availability of the RNs. The DS-DAT is a complex measure and we did provide raters with detailed instructions. Moreover, this limitation does not seem to pose a threat to our conclusions since the results confirm our hypotheses and are consistent with those of other researchers.

Dementia creates a paradoxical context in which patients are more vulnerable to various sources of discomfort while, at the same time, being less able to modify these by themselves or to communicate their discomfort directly to their caregivers. Further understanding of the internal determinants of behavioral symptoms in dementia will ultimately lead to finer assessment and more effective treatment of patients.

## Conclusion

Our findings provide further evidence of the association between discomfort and agitation in persons with dementia and reveal that this association is particularly strong for VAB and NAPB.

## Competing interests

The author(s) declare that they have no competing interests.

## Authors' contributions

ICP conceived and carried out the study and drafted the manuscript. PL contributed to the conception of the study and helped to draft the manuscript. Both authors read and approved the final manuscript.

## Pre-publication history

The pre-publication history for this paper can be accessed here:


